# Supplementary material for: GHz-rate optical phase shift in light-matter interaction-engineered, silicon-ferroelectric nematic liquid crystals
Source: Nat Commun. 2025 Oct 7;16:8902. doi: 10.1038/s41467-025-63924-y (PMC12504419; doi:10.1038/s41467-025-63924-y)
Supplement: Supplementary file 1 — Supplementary Information [file 41467_2025_63924_MOESM1_ESM.pdf]

Supplementary Information

GHz-rate optical phase shift in light matter interaction-engineered, silicon-ferroelectric nematic liquid crystals

Iman Taghavi[1,2*], Omid Esmaeeli[1], Sheri Jahan Chowdhury[1], Kashif Masud Awan[3], Mustafa Hammood[1,2], Matthew Mitchell[1,3], Donald Witt[1,3], Cory Pecinovsky[4], Jason Sickler[4], Jeff F. Young[1,3,5], Nicolas A.F. Jaeger[1], Sudip Shekhar[1,2,6*], and Lukas Chrostowski[1,2,3*]

[1]Department of Electrical and Computer Engineering, University of British Columbia, 2332 Main Mall, Vancouver, V6T 1Z4, B.C., Canada.

[2]Dream Photonics, 2366 Main Mall, Vancouver, V6T 1Z4, B.C., Canada.

[3]Quantum Matter Institute, University of British Columbia, 2355 E Mall, Vancouver, V6T 1Z4, B.C., Canada.

[4]Polaris Electro-Optics, 3400 Industrial Ln, 3130 25th St, Broomfield, 80020, CO, USA.

[5]Department of Physics and Astronomy, University of British Columbia, 6224 Agricultural Road, Vancouver, V6T 1Z1, B.C., Canada.

[6]Dream Photonics Inc., Woodinville, WA, USA.

*Corresponding author(s). E-mail(s): staghavi3@ece.ubc.ca; sudip@ece.ubc.ca; lukasc@ece.ubc.ca;

Supplementary Note 1: Pockels coefficient extraction

The derivation of an analytical relationship between S_{21}, the device characteristics and setup parameters based on Fig. 1 is as follows [1]:

$$S_{21} = 10 \times \log\left[\frac{\partial P_{\text{in,VNA}}}{\partial P_{\text{out,VNA}}}\right] = 20 \times \log\left[\frac{\partial V_{\text{in,VNA}}}{\partial V_{\text{out,VNA}}}\right] \text{ ; since the } Z_{\text{in}} = Z_{\text{out}} = 50\ \Omega \quad (1)$$

$$= 20 \times \log\left[\frac{\partial V_{\text{out,LNA}}}{\partial V_{\text{in,amp}}}\right] = 20 \times \log\left[\frac{\partial V_{\text{out,det}} \times G_{\text{RF},2}}{\partial V_{\text{in,MZM}}/G_{\text{RF},1}}\right] \text{ ; since } G_{\text{RF},1} \times G_{\text{RF},2} = G_{\text{RF}} \quad (2)$$

$$= 20 \times \log\left[\frac{\partial V_{\text{out,det}} \times G_{\text{RF}}}{\partial V_{\text{in,MZM}}} \times \frac{\partial P_{\text{in,det}}}{\partial P_{\text{in,det}}}\right] \text{ ; introduce } \partial P_{\text{in,det}} \quad (3)$$

$$= 20 \times \log\left[\frac{\partial V_{\text{out,det}}}{\partial P_{\text{in,det}}} \times \frac{\partial P_{\text{out,MZM}}}{\partial V_{\text{in,MZM}}} \times G_{\text{RF}}\right] \text{ ; since } P_{\text{in,det}} = P_{\text{out,MZM}} \quad (4)$$

$$= 20 \times \log\left[\frac{\partial V_{\text{out,det}}}{\partial P_{\text{in,det}}} \times \frac{\partial P_{\text{out,MZM}}}{\partial V_{\text{in,MZM}}} \times G_{\text{RF}} \times \frac{\partial n}{\partial n}\right] \text{ ; introduce } \partial n \quad (5)$$

$$= 20 \times \log\left[\frac{\partial V_{\text{out,det}}}{\partial P_{\text{in,det}}} \times \frac{\partial P_{\text{out,MZM}}}{\partial n} \times \frac{\partial n}{\partial V_{\text{in,MZM}}} \times G_{\text{RF}}\right] \text{ ; introduce } \partial n \quad (6)$$

$$= 20 \times \log\left[\frac{\partial V_{\text{out,det}}}{\partial P_{\text{in,det}}} \times \frac{\partial P_{\text{in,MZM}} \times \alpha}{\partial n} \times \frac{\partial n}{\partial V_{\text{in,MZM}}} \times G_{\text{RF}}\right] \text{ ; since } P_{\text{out,MZM}} = P_{\text{in,MZM}} \times \alpha \quad (7)$$

$$= 20 \times \log\left[\frac{\partial V_{\text{out,det}}}{\partial P_{\text{in,det}}} \times \frac{\partial P_{\text{in,MZM}}}{\partial n} \times \frac{\partial n}{\partial V_{\text{in,MZM}}} \times G_{\text{RF}} \times \alpha\right] \quad (8)$$

Three of the factors (i.e., $[\partial P_{\text{in,MZM}}/\partial n] \times [\partial n/\partial V_{\text{in,MZM}}] \times \alpha$) are the device-related parameters. In contrast, the remaining two (i.e., $[\partial V_{\text{out,det}}/\partial P_{\text{in,det}}] \times G_{\text{RF}}$) are setup-related.

In the third step, we introduced ∂n as opposed to $\partial \lambda$ suggested by [1]. In a Mach-Zehnder Modulator (MZM), $\partial n/\partial V$ is connected to various device and material parameters [2, 3]. For resonant modulators, $\partial \lambda/\partial V$ is a more intuitive figure of merit to experiment with [4]. Knowing the modulator's transfer function allows an easy conversion between the two measurements. For an MZM, the half-wave voltage (in push-pull configuration) can be defined as

$$V_\pi = \frac{FSR}{4 \times \partial \lambda/\partial V} \quad (9)$$

Also, according to Eq. (2) in the manuscript,

$$V_\pi = \frac{\lambda}{2L \times \partial n/\partial V} \quad (10)$$

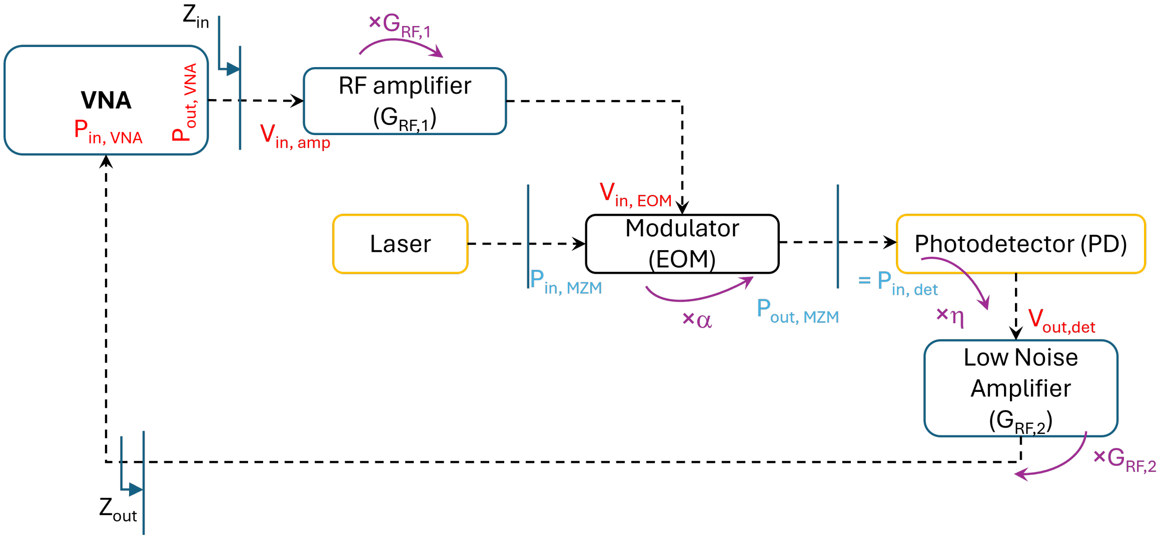

Supplementary Figure 1 - AC Characterization block diagram. The Pockels coefficient (r_{33}) in an electro-optic modulator (EOM) can be extracted from S_{21} provided by a vector network analyzer (VNA).

Therefore, we have:

$$\frac{\partial n}{\partial V} = \frac{2 \times \lambda \left[\partial\lambda / \partial V \right]}{FSR \times L} \tag{11}$$

where L is the length of the MZM arm (i.e., active phase shifter), FSR is the free-spectral range of the optical spectrum, λ is the operating wavelength, and ∂V is the applied voltage to alter the refractive index by ∂n.

Supplementary Note 2: Calculation of the optical confinement and field overlap integral

To compare the finger-loaded strip (FLS) waveguide to equivalent slotted and non-slotted structures, optical mode confinement ($\mathbf{E}_x$) is estimated in the light-matter interaction (LMI) zones, as shown in Fig. 2. Ignoring the weak mode that leaked onto the non-slotted waveguide, two identical zones can be seen around the sidewalls. Reducing the waveguide width to 300 nm, which matches the core width in the FLS structure (i.e., W_c), improves mode confinement.

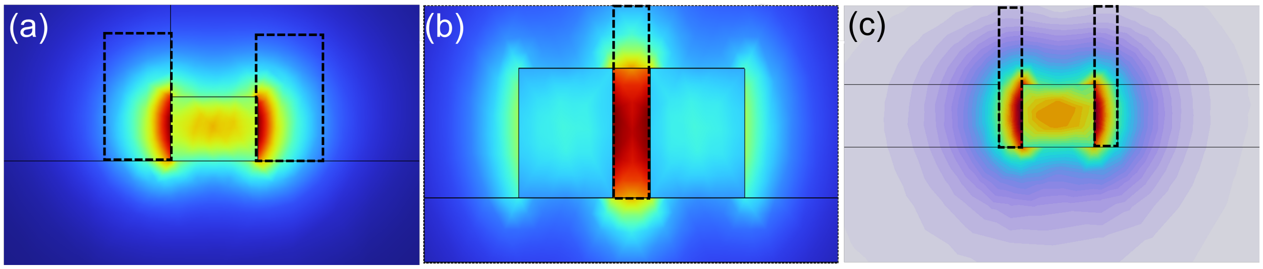

Supplementary Figure 2 - Optical confinement factor (β) and field overlap integral (Γ). The optical mode confined in the light-matter interaction regions shown by the dotted regions in (a) non-slotted, (b) slotted, and (c) finger-loaded strip waveguide architectures.

Despite this, the waveguide exhibits a low overlap integral (Γ) between optical ($\mathbf{E}_x$) and electrical fields ($\mathbf{E}_e$), as examined by:

$$\Gamma = \frac{1}{Z_0} \iiint\limits_V n_{\text{eff}}(\mathbf{E}_{\text{e}}) \left|\mathbf{E}_{\text{x}}\right|^2 / \iint\limits_A L \times \text{Re}(\mathbf{E} \times \mathbf{H}^*) \tag{12}$$

In Equation 12, n_{eff} is the field-dependent effective mode index of FLS waveguide, A and V are the cross-section and volume of the LMI region, Z_0 is the free space impedance, and $\mathbf{H}$ and $\mathbf{E}$ are the magnetic and electric fields of the optical mode, respectively. The denominator is proportional to the total optical power supplied into the waveguide.

Slot waveguides provide significant confinement for $\mathbf{E}_x$ due to the electric field discontinuity at slot interfaces. The transverse electric (TE) field within the slot (i.e., in the polymer-infiltrated region represented by the dotted region in Fig. 2(b)) will be augmented by $n_{\text{Si}}^2/n_{\text{FN-LC}}^2$ ($\approx 3.42^2/1.72^2 = 3.95$), where n_{Si} and $n_{\text{FN-LC}}$ are the silicon and ferroelectric nematic liquid crystal (FN-LC) materials, respectively. Another advantage of the slot waveguide is the electrical field confinement ($\mathbf{E}_e$) achieved by the narrow slot. As a result of the slot waveguide's double confinement, Γ is maximized. An FLS waveguide, on the other hand, features a double-slot region on both sides of the core, allowing for moderate mode confinement. Our simulations indicate that if correctly doped, Γ in FLS can reach the same level as in slot waveguides. Finally, the modulation efficiency is dependent on Γ/d_{eff}, as explained in the study.

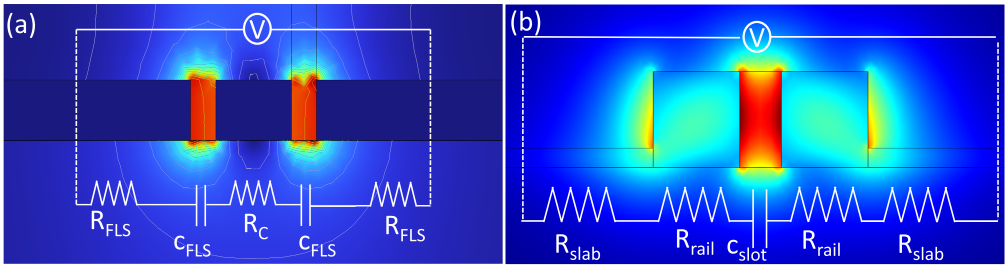

Supplementary Figure 3 - Equivalent RC circuit of (a) a finger-loaded strip (FLS) and (b) an equivalent strip-loaded slot waveguide. The cross-section of the FLS waveguide shows the resistance associated with the finger and core and the capacitance of the two gaps between the finger and core. For the case of the slot waveguide, we assume $R_{\text{slab}} >> R_{\text{rail}}$.

Supplementary Note 3: Equivalent RC circuit

Figure 3(a) shows a more accurate equivalent circuit for the FLS waveguide, which includes the effect of resistance associated with the undoped core waveguide (R_{C}). The resulting RC time constant would be equal to:

$$\tau_{\text{FLS}} \approx \left[2\times \ R_{\text{FLS}} + R_{\text{C}}\right] \times \ \left[\frac{C_{\text{FLS}}}{2}\right] \tag{13}$$

For the device reported in this study, our simulations indicated $R_{\text{C}} \approx 0.063\ R_{\text{FLS}}$, which can be ignored. Figure 3(b) shows the corresponding RC circuit for a slot waveguide with a slot width of $W_{\text{slot}} = d_{\text{f-c}}$, where $d_{\text{f-c}}$ represents the gap between the fingertip and core in the FLS waveguide. Accordingly,

$$\tau_{\text{slot}} \approx \left[2\times \ (R_{\text{slab}} + R_{\text{rail}})\right] \times \ \left[C_{\text{slot}}\right] \approx 2R_{\text{slab}}C_{\text{slot}} \tag{14}$$

Furthermore, we know that

$$\frac{R_{\text{FLS}}}{R_{\text{slab}}} \approx \left[\frac{V_{\text{FLS}}}{V_{\text{slab}}}\right]^{-1} \approx \ \left[\frac{h_{\text{fe}}}{h_{\text{pe}}} \times \ \gamma_{\text{FLS}}\right]^{-1} \tag{15}$$

where h_{fe} and h_{pe} are the full-etch and partial-etch silicon heights, what γ_{FLS} is the duty cycle of the fingers in the FLS waveguide, and V_{FLS} and V_{slab} are the total volumes of silicon

fingers versus those of silicon slabs and rails in slot waveguides, respectively. Our simulations demonstrated that for an equal doping concentration, one may conclude

$$R_{\text{FLS}}/R_{\text{slab}} \approx 0.48 \tag{16}$$

We also know that

$$C_{\text{slot}}/C_{\text{FLS}} \approx \; W_{\text{slot}}/d_{\text{f-c}} \equiv 1 \tag{17}$$

To optimize the RF-optical loss trade-off, at least one degree of doping in the order of $\sim 10^{17}$ cm^{-3} is necessary, implying $R_{\text{C}} \approx \; R_{\text{FLS}}$. This results in

$$\tau_{\text{FLS}} \approx \left[3 \times \; R_{\text{FLS}}\right] \times \; \left[C_{\text{FLS}}/2\right] \tag{18}$$

Based on Equations 15-18, one can conclude that $\tau_{\text{FLS}} = 0.36 \times \tau_{\text{slot}}$. Equivalently, one might theoretically leverage this reduced τ_{FLS} towards less doping in fingers to achieve the same τ, resulting in a smaller overall $V\pi L\alpha$ for the FLS waveguide compared to slot waveguides.

Supplementary Note 4: Device and Performance Optimization

Modifying several aspects of the device may enhance its performance beyond what is demonstrated in the present work, as outlined below:

Device properties- To begin, reducing the equivalent resistance of the silicon fingers responsible for transmitting the RF signal through the periodic structuredenoted as R_{FLS} is essential. These fingers are integrated into the silicon pedestal, beneath which the metal electrodes are recessed. Our simulations indicate that employing a two-step boron (p-type) ion implantation process significantly decreases R_{FLS}, as detailed in Table 1. We adopted a doping profile consistent with those used in similar strip-loaded slot waveguides, as reported in [5].

Furthermore, it is worth considering structural modifications to the FLS waveguide, as illustrated in Fig. 4. To prevent any unintended variations in propagation losswhich are challenging to model accuratelywe maintained several geometric parameters: the waveguide core width (W_{c}), the finger width (L_{f}), and the duty cycle of the periodic FLS waveguide ($L_{\text{g}}/(L_{\text{g}} + L_{\text{f}})$).

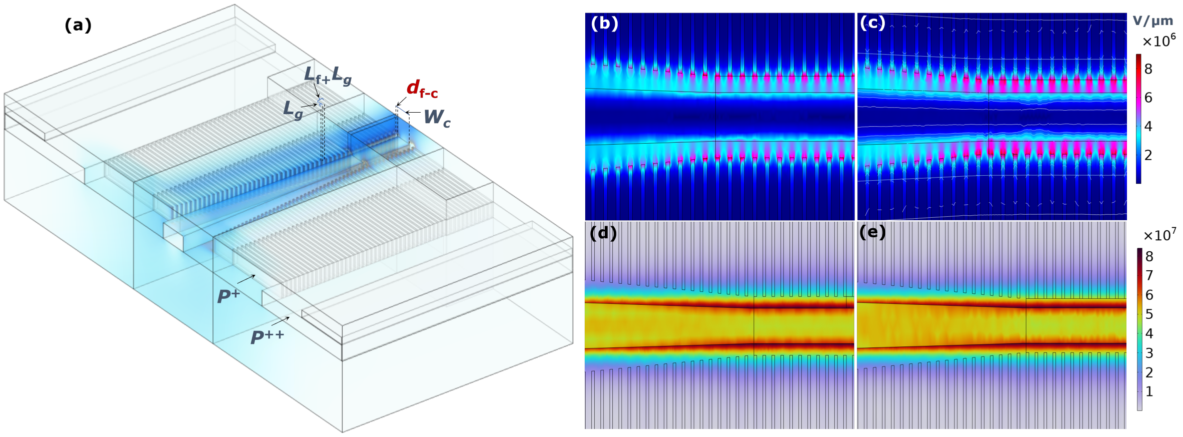

Supplementary Figure 4 - Device improvement. (a) 3D illustration of the finger-loaded strip (FLS) structure with the chosen structural factor to modify ($d_{\text{f-c}}$ reduction from 100 nm to 80 nm) while keeping others intact (e.g., W_{c}, L_{f}, and L_{g}). The device is implanted with p-type dopant at two different levels denoted by p^{+} and p^{++} specified in Table 1. The electrical field ($\mathbf{E}_{\text{e}}$) in the y-slice perspective (y = 110 nm) of the (b) original and (c) optimized design shows an increase of the average field from ≈ 5 Vμm^{-1} to ≈ 6 Vμm^{-1}. The transverse electric optical mode profile ($\mathbf{E}_{\text{x}}$) in the y-slice perspective (y = 110 nm) of the (d) original and (e) optimized design does not noticeably change; thus, no significant change in optical mode interaction with the periodic FLS structure and hence optical loss is expected.

Simulation results suggest that reducing the finger-to-core spacing ($d_{\text{f-c}}$) can significantly improve the device factor $\Gamma \times \alpha/d_{\text{eff}}$ through multiple mechanisms: a) It decreases the effective electrode spacing (d_{eff}) (refer to Methods and the caption of Fig. 2 of the main text). b) It marginally increases the overlap integral between the electrical and optical modal fields (Γ). c) Given that the optical interaction with the periodic structure remains constant, the propagation loss is expected to remain unchanged.

According to Equations (2-3) in the manuscript, it is well understood that enhancing $\Gamma \times \alpha/d_{\text{eff}}$ directly improves the modulation sensitivity ($\partial n/\partial V$), which, in turn, enhances the $V_{\pi}L$ performance. A minor drawback of decreasing $d_{\text{f-c}}$ is a slight increase in the equivalent capacitance of the double-slot-like FLS structure (C_{FLS}).

Material properties- Analogous to electro-optic (EO) polymers, newly synthesized FN-LC materials incorporating a higher concentration of dye molecules [6] or possessing intrinsically large molecular hyperpolarizabilities demonstrate enhanced EO performance, as indicated by an increased Pockels coefficient (i.e., $n^3 r_{33}$). Our co-authors (at Polaris Electro-Optics, Inc.)

	Current design	Improved design
**Si doping [cm^{-3}]**	5.0×10^{14}	P^{+} 4.0×10^{17} (P^{++} 1×10^{19}) for fingers (pedestal)
$d_{\textbf{f-c}}$ **[nm]**	100	80
$d_{\textbf{eff}}$ **[nm]**	200	167
Γ	0.26	0.31
α **[dBmm^{-1}]**	5.2	5.33
r_{33} **[pmV^{-1}]**	9.5	50
$R_{\textbf{FLS}}$ [Ω]	820.7	28.73
$C_{\textbf{FLS}}$ **[fF]**	70.29	87.86
$1/(2\pi R_{\textbf{FLS}} C_{\textbf{FLS}})$ [s^{-1}]	2.76	63.05
$V_\pi L$ **[V·mm]**	25.7	3.36

Supplementary Table 1 - Device structure and material property improvement

now have a next-generation FN-LC material with an estimated $r_{33} \approx 50$ pmV^{-1} (unpublished), offering a promising pathway to further improve the $V_\pi L$ metric.

Table 1 summarizes the proposed device and material enhancements. Green and red indicators denote whether each modification is expected to enhance or degrade performance, respectively. The three straightforward adjustments discussed previously lead to an increase in EO bandwidth (see Fig. 5(a)) and an improvement in $V_\pi L$, without introducing significant additional insertion loss. We benchmarked both the original and optimized designs against a previously reported FN-LC device employing a slot waveguide architecture [7], along with several other leading-edge technologies, as shown in Fig. 5(b). While this comparison does not represent an exhaustive survey of all record-setting modulators across various material platforms, we focused on those exhibiting the highest $f_{-3\text{dB}}/V_\pi L$ figures of merit, as indicated by the contour lineswhere $f_{-3\text{dB}}$ refers to the -3 dB EO bandwidth. When infiltrated with the newly developed FN-LC formulations, the enhanced FLS waveguides demonstrate the potential to compete with existing technologies that are typically more complex and costly to fabricate.

Supplementary Note 5: Optical Sideband Measurements

As a secondary proof of EO activity in the material, we initiated measurements based on the optical sideband method. In conjunction with the non-zero S_{21} data presented in Fig. 5(a), the measurement is intended to provide further evidence supporting the existence of the rapid phase shift mechanism proposed in the current work for FN-LC. In other words, the device would have ceased to respond at frequencies beyond 100 kHz, a threshold at which conventional paraelectric nematic liquid crystal (PN-LC) devices are known to fail [16].

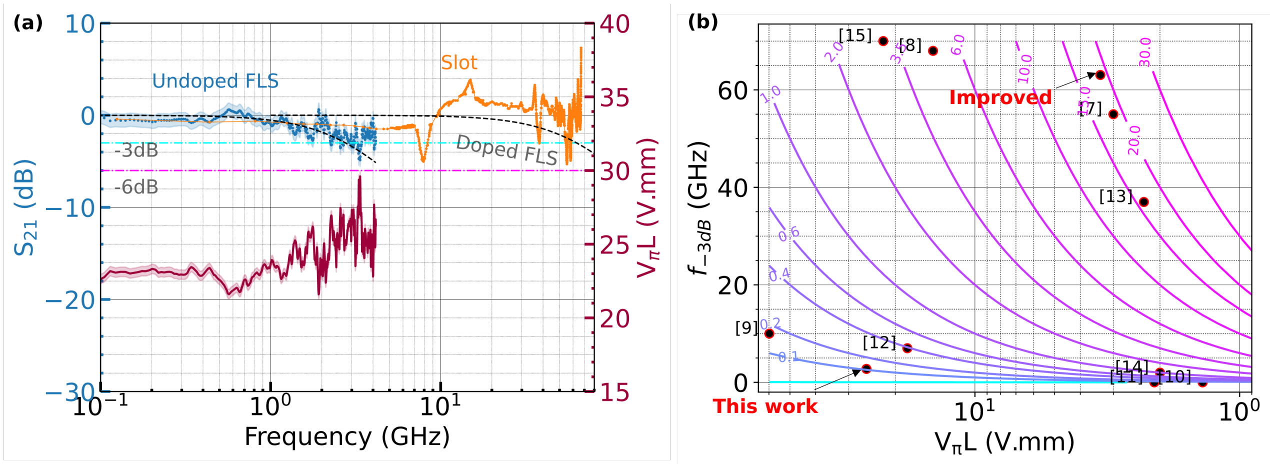

Supplementary Figure 5 - Performance enhancements and comparisons. (a) Measured $|S_{21}|$ of the undoped finger-loaded strip (FLS) described in this study, coupled with the post-processed AC values of $V_\pi L$ and r_{33}. To demonstrate the doping effect, measured $|S_{21}|$ data of a doped slot waveguide [7] and simulation results of the enhanced FLS waveguide are superimposed. All three are coated with a ferroelectric nematic liquid crystal (FN-LC). (b) A quantitative comparison between this work, in both current and improved formats, slotted waveguide FN-LC [7] and some other conventional phase shifter technologies, including polymer [8, 9], thermo-optic (doped Si) [10] and (metal) [11], *pn*-Si [12, 13], barium titanate [14], lithium niobate [15] (contour lines: $f_{\text{-3dB}}/V_\pi L$ [GHz(V.mm)$^{-1}$]).

Specifically, we utilized an optical spectrum analyzer (Ando-AQ6317B) in combination with a synthesizer (HP-8657A) to perform the recommended sideband measurements, as illustrated in Fig. 6. An RF power splitter (Mini-Circuits ZFSCJ-2-1), which introduces a 180-degree phase difference between the two output ports, was used to drive each arm. Concurrently, we applied two different polarities of DC voltage to each arm to ensure optimal dipole alignment and to operate the MZM in push-pull mode. When a sinusoidal signal at frequency f_{m} is used to modulate an optical carrier at frequency f_{c}, the MZM's optical output field (E_{o}) can be derived as described in [17].

$$E_{\text{o}}(t) = \frac{E_{\text{i}}}{2}\Big[\exp(j[2\pi f_{\text{c}}t + \phi_1(t)]) + \exp(j[2\pi f_{\text{c}}t + \phi_2(t)])\Big] \tag{19}$$

where E_{i} is the complex field of the input optical signal, ϕ_1 and ϕ_2 are the phase shifts associated with each arm. The voltage applied to the two arms is $v_1(t) = V_{\text{DC,1}} + V_{\text{AC}} \times \cos(2\pi f_{\text{m}}t + \theta)$ and $v_2(t) = V_{\text{DC,2}} + V_{\text{AC}} \times \cos(2\pi f_{\text{m}}t)$. Accordingly, $\phi_1(t) = \phi_{0,1} + \beta_1\cos(2\pi f_{\text{m}}t)$ and $\phi_2(t) = \phi_{0,2} + \beta_2\cos(2\pi f_{\text{m}}t)$ are the modulation indices associated with each arm, where $\beta_{\text{i}} = \pi V_{\text{AC}}/V_{\pi,\text{i}}$ $(i = 1, 2)$, $\phi_{0,1} = \pi V_{\text{DC,1}}/V_{\pi,1} + 2\pi\left[\Delta n L/\lambda\right]$ and $\phi_{0,2} = \pi V_{\text{DC,2}}/V_{\pi,2}$ are

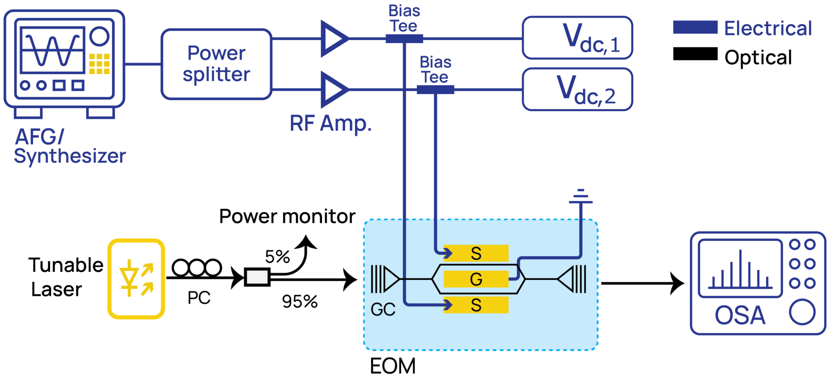

Supplementary Figure 6 - Experimental setup for sideband measurements. An optical spectrum analyzer (OSA) and an arbitrary function generator(AFG)/synthesizer is used to characterize the electro-optic modulator (EOM). The two DC voltage sources are acquired to provide dipole alignment and drive the modulator at the quadrature point.

the static phases in each arm, ΔL is the path length difference between the two arms, n is the effective index of refraction, and λ is the operation wavelength. In its simplified format, $\phi_{0,1} - \phi_{0,2} = \phi_0$ and $\beta_1 = \beta_2 = \pi V_{\mathrm{AC}}/V_\pi$, thus, the Equation 19 can then be rewritten as [17]

$$E_{\mathrm{o}}(t) = \frac{E_{\mathrm{i}} e^{j2\pi f_{\mathrm{c}} t}}{2} \left[exp(j[\phi_0 + \beta \cos(2\pi f_{\mathrm{m}} t + \theta)]) + \exp(j[\beta \cos(2\pi f_{\mathrm{m}} t)]) \right] \tag{20}$$

Based on Fig. 3(b) in the main text, a DC voltage of $V_{\mathrm{DC}} = V_{\mathrm{align}} = 2\ V$ is enough to fully orient dipoles in each arm. In addition to that, we need to adjust $V_{\mathrm{DC},1}$ to bias the MZM at the quadrature point so that $\phi_0 = 3\pi/2$. It results in $e^{j\phi_0} = -j$, hence, Equation 20 becomes

$$E_{\mathrm{o}}(t) = \frac{E_{\mathrm{i}} e^{j2\pi f_{\mathrm{c}} t}}{2} \left[\exp(j[\beta \cos(2\pi f_{\mathrm{m}} t)]) - j\exp(j[\beta \cos(2\pi f_{\mathrm{m}} t + \theta)]) \right] \tag{21}$$

Knowing that $\theta \approx 180^\circ$ is mainly controlled by the power splitter, we have

$$E_{\mathrm{o}}(t) = \frac{E_{\mathrm{i}} e^{j2\pi f_{\mathrm{c}} t}}{2} \left[\exp(j[\beta \cos(2\pi f_{\mathrm{m}} t)]) - j\exp(-j[\beta \cos(2\pi f_{\mathrm{m}} t)]) \right] \tag{22}$$

On the other hand, we know [17]

$$\exp(j[\beta\cos(2\pi f_{\rm m}t)]) = \sum_{k=-\infty}^{\infty} j^k J_k(\beta) e^{2jk\pi f_{\rm m}t} \tag{23}$$

Considering that $J_{-k}(\beta) = (-1)^k J_k(\beta)$, Equation 22 simplifies to [17]

$$E_{\rm o}(t) = \frac{E_{\rm i}e^{j2\pi f_{\rm c}t}}{2}\big[... + \big(1+j\big)J_1(\beta)e^{-2jk\pi f_{\rm m}t} + \big(1-j\big)J_0(\beta) + \big(1+j\big)J_1(\beta)e^{2jk\pi f_{\rm m}t} + ...\big] \tag{24}$$

which determines the output power at the carrier frequency and harmonics as [17]

$$|\frac{E_{\rm o}(t)}{E_{\rm i}(t)}|^2 = \frac{1}{2} \times \begin{cases} J_0^2(\beta) & ;\text{carrier frequency} \\ J_1^2(\beta) & ;\text{ 1st harmonic (right and left sidebands) } @f_c \pm f_m \\ J_2^2(\beta) & ;\text{ 2nd harmonic (right and left sidebands) } @f_c \pm 2f_m \\ ... \end{cases} \tag{25}$$

Figure 6(a) shows the optical output spectra of the MZM driven with $f_{\rm m} = 0.5$ GHz and the fitted data using the Bessel function summarized in Equation 24. The difference between the carrier and the first sidebands ($\Delta y_1 \propto \big[J_0^2(\beta)/J_1^2(\beta)\big]_{\rm dB} \approx 12$ dB), which, according to 7-(b), is related to $\beta = \pi V_{\rm AC}/V_\pi \approx 0.49$. It provides an estimate of $V_\pi \approx 51\ V$, which is the same as what we found using the $S-$parameter method and presented in Table 2 of the main text. The slight variation between the two sidebands (Δy_2) is likely due to device asymmetry or the driving levels, as follows:

- The RF routes, including wire bonds employed to drive the MZM in the push-pull arrangement, are not the same, hence $\theta \neq 180$.
- The tiny variation in how dipoles infiltrate and position themselves in the two arms in response to $V_{\rm DC}$ causes $V_{\pi,1} \neq V_{\pi,2}$.
- Similar to EO polymers, we also expect that $V_{\pi,1} \neq V_{\pi,2}$ since the RF field is in parallel to the alignment in one arm anti-parallel in the other arm [18].

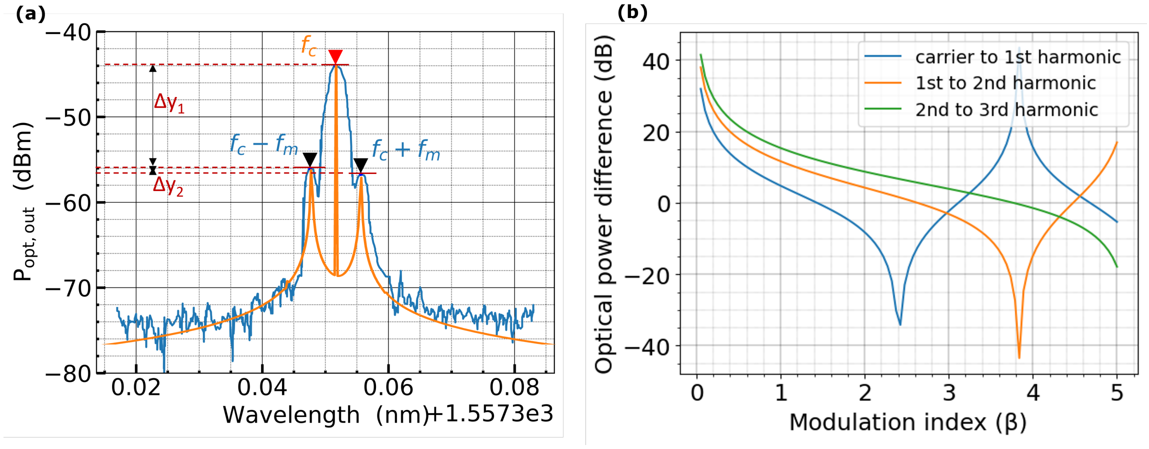

Supplementary Figure 7 - Sideband measurements. (a) Power spectral density of a double-sideband modulated optical signal (at a carrier frequency of f_c) using a sinusoidal signal (at a frequency of f_m) driving the MZM in push-pull configuration. Optical amplitude difference of the carrier-to-1st and carrier-to-2nd harmonics, denoted by Δy_1 and Δy_2, respectively, can be used to estimate half-wave voltage (V_π). (b) Optical amplitude difference between the carrier and harmonics as a function of the modulation index ($\beta = \pi V_\mathrm{AC}/V_\pi$).

References

[1] Gould, M., Baehr-Jones, T., Ding, R., Huang, S., Luo, J., Jen, A.K.-Y., Fedeli, J.-M., Fournier, M., Hochberg, M.: Silicon-polymer hybrid slot waveguide ring-resonator modulator. Optics express **19**(5), 3952–3961 (2011)

[2] Taghavi, I., Dehghannasiri, R., Fan, T., Tofini, A., Moradinejad, H., Efterkhar, A.A., Shekhar, S., Chrostowski, L., Jaeger, N.A., Adibi, A.: Enhanced poling and infiltration for highly efficient electro-optic polymer-based mach-zehnder modulators. Optics Express **30**(15), 27841–27857 (2022)

[3] Palmer, R., Koeber, S., Elder, D.L., Woessner, M., Heni, W., Korn, D., Lauermann, M., Bogaerts, W., Dalton, L., Freude, W., *et al.*: High-speed, low drive-voltage silicon-organic hybrid modulator based on a binary-chromophore electro-optic material. Journal of Lightwave Technology **32**(16), 2726–2734 (2014)

[4] Brosi, J.-M., Koos, C., Andreani, L.C., Waldow, M., Leuthold, J., Freude, W.: High-speed low-voltage electro-optic modulator with a polymer-infiltrated silicon photonic crystal waveguide. Optics Express **16**(6), 4177–4191 (2008)

[5] Zwickel, H., Singer, S., Kieninger, C., Kutuvantavida, Y., Muradyan, N., Wahlbrink, T., Yokoyama, S., Randel, S., Freude, W., Koos, C.: Verified equivalent-circuit model for slot-waveguide modulators. Optics express **28**(9), 12951–12976 (2020)

[6] Chen, X., Patel, C., Bradfield, A., Sickler, J.W., Maclennan, J.E., Clark, N.A., Glaser, M.A., Pecinovsky,

C.: Ferroelectric nematic materials for high-speed electro-optic applications. In: Liquid Crystals Optics and Photonic Devices, vol. 13016, pp. 24–29 (2024). SPIE

[7] Onural, D., Wang, I., Chiang, L.-Y., Raja, S., Zhang, X., Singh, M., Li, D., Dao, H., Pajk, S., Sickler, J.W., *et al.*: Hybrid integration of silicon slot photonics with ferroelectric nematic liquid crystal for poling-free pockels-effect modulation. In: 2024 Conference on Lasers and Electro-Optics (CLEO), pp. 1–2 (2024). IEEE

[8] Lu, G.-W., Hong, J., Qiu, F., Spring, A.M., Kashino, T., Oshima, J., Ozawa, M.-a., Nawata, H., Yokoyama, S.: High-temperature-resistant silicon-polymer hybrid modulator operating at up to 200 gbit s- 1 for energy-efficient datacentres and harsh-environment applications. Nature communications **11**(1), 1–9 (2020)

[9] Qiu, F., Sato, H., Spring, A.M., Maeda, D., Ozawa, M.-a., Odoi, K., Aoki, I., Otomo, A., Yokoyama, S.: Ultra-thin silicon/electro-optic polymer hybrid waveguide modulators. Applied Physics Letters **107**(12), 92–1 (2015)

[10] Geis, M.W., Spector, S.J., Williamson, R., Lyszczarz, T.: Submicrosecond submilliwatt silicon-on-insulator thermooptic switch. IEEE photonics technology letters **16**(11), 2514–2516 (2004)

[11] Espinola, R., Tsai, M., Yardley, J.T., Osgood, R.: Fast and low-power thermooptic switch on thin silicon-on-insulator. IEEE Photonics Technology Letters **15**(10), 1366–1368 (2003)

[12] Park, J.W., You, J.-B., Kim, I.G., Kim, G.: High-modulation efficiency silicon mach-zehnder optical modulator based on carrier depletion in a pn diode. Optics express **17**(18), 15520–15524 (2009)

[13] Mishra, D., Sonkar, R.K.: Analysis of germanium-doped silicon vertical pn junction optical phase shifter. Journal of the Optical Society of America B **36**(5), 1348–1354 (2019)

[14] Eltes, F., Mai, C., Caimi, D., Kroh, M., Popoff, Y., Winzer, G., Petousi, D., Lischke, S., Ortmann, J.E., Czornomaz, L., *et al.*: A batio 3-based electro-optic pockels modulator monolithically integrated on an advanced silicon photonics platform. Journal of Lightwave Technology **37**(5), 1456–1462 (2019)

[15] He, M., Xu, M., Ren, Y., Jian, J., Ruan, Z., Xu, Y., Gao, S., Sun, S., Wen, X., Zhou, L., *et al.*: High-performance hybrid silicon and lithium niobate mach–zehnder modulators for 100 gbit s- 1 and beyond. Nature Photonics **13**(5), 359–364 (2019)

[16] Xing, Y., Ako, T., George, J.P., Korn, D., Yu, H., Verheyen, P., Pantouvaki, M., Lepage, G., Absil, P., Ruocco, A., *et al.*: Digitally controlled phase shifter using an soi slot waveguide with liquid crystal infiltration. IEEE Photonics Technology Letters **27**(12), 1269–1272 (2015)

[17] Hui, R.: Introduction to Fiber-optic Communications. Academic Press, ??? (2019)

[18] Takayesu, J., Hochberg, M., Baehr-Jones, T., Chan, E., Wang, G., Sullivan, P., Liao, Y., Davies, J., Dalton, L., Scherer, A., *et al.*: A hybrid electrooptic microring resonator-based 1 x 4 x 1 roadm for wafer scale optical interconnects. Journal of lightwave technology **27**(4), 440–448 (2009)
